# Development of a culturally appropriate computer-delivered tailored internet-based health literacy intervention for spanish-dominant hispanics living with HIV

**DOI:** 10.1186/s12911-014-0103-9

**Published:** 2014-11-30

**Authors:** Robin J Jacobs, Joshua Caballero, Raymond L Ownby, Michael N Kane

**Affiliations:** Biomedical Informatics/Psychiatry and Behavioral Medicine College of Osteopathic Medicine Nova Southeastern University 3200 S, University Drive Terry Building HPD/OST, Fort Lauderdale, FL 33328 USA; College of Pharmacy, Nova Southeastern University, Fort Lauderdale, FL 33328 USA; Psychiatry and Behavioral, Medicine College of Osteopathic Medicine Nova Southeastern University, Fort Lauderdale, FL 33328 USA; College for Design & Social Inquiry School of Social Work Florida, Atlantic University, Glades road, Boca Raton, FL 33431 777 USA

**Keywords:** eHealth, Consumer health informatics, Health literacy, HIV/AIDS, Internet, Hispanics/Latinos, Tailored intervention, Medication adherence, Health technology, Ethnic minority, Health disparities

## Abstract

**Background:**

Low health literacy is associated with poor medication adherence in persons with human immunodeficiency virus (HIV), which can lead to poor health outcomes. As linguistic minorities, Spanish-dominant Hispanics (SDH) face challenges such as difficulties in obtaining and understanding accurate information about HIV and its treatment. Traditional health educational methods (e.g., pamphlets, talking) may not be as effective as delivering through alternate venues. Technology-based health information interventions have the potential for being readily available on desktop computers or over the Internet. The purpose of this research was to adapt a theoretically-based computer application (initially developed for English-speaking HIV-positive persons) that will provide linguistically and culturally appropriate tailored health education to Spanish-dominant Hispanics with HIV (HIV + SDH).

**Methods:**

A mixed methods approach using quantitative and qualitative interviews with 25 HIV + SDH and 5 key informants guided by the Information-Motivation-Behavioral (IMB) Skills model was used to investigate cultural factors influencing medication adherence in HIV + SDH. We used a triangulation approach to identify major themes within cultural contexts relevant to understanding factors related to motivation to adhere to treatment. From this data we adapted an automated computer-based health literacy intervention to be delivered in Spanish.

**Results:**

Culture-specific motivational factors for treatment adherence in HIV + SDH persons that emerged from the data were stigma, *familismo* (family), mood, and social support. Using this data, we developed a culturally and linguistically adapted a tailored intervention that provides information about HIV infection, treatment, and medication related problem solving skills (proven effective in English-speaking populations) that can be delivered using touch-screen computers, tablets, and smartphones to be tested in a future study.

**Conclusion:**

Using a theoretically-grounded Internet-based eHealth education intervention that builds on knowledge and also targets core cultural determinants of adherence may prove a highly effective approach to improve health literacy and medication decision-making in this group.

## Background

The link between limited health literacy (HL) and poor health has been well documented. In 2004, both the Agency for Healthcare Research and Quality and the Institute of Medicine published reports on HL and health outcomes. Both reports concluded that limited HL is negatively associated with the use of preventive services (e.g., mammograms), management of chronic conditions (e.g., diabetes) and self-reported health. High levels of adherence to antiretroviral (ARV) medications are linked to better clinical outcomes [1] and reduced risk of viral resistance to medications [2]. Failure to maintain optimal levels of adherence is associated with drug resistance and treatment failure. Additional studies have linked limited HL to medication dosing errors and mortality [3]. Given persons in ethnic and racial minority groups have lower levels of HL and the association of HL with health, HL may be a factor in race and ethnicity-related health disparities [4,5].

### Importance of medication adherence

High levels of adherence to antiretroviral (ARV) medications are linked to better clinical outcomes [6] and reduced risk of viral resistance to medications [7]. Further, persons with high adherence are more likely to have low levels of virus. Low viral loads are associated with reduced risk of transmitting the virus to others [8,9]. Adherence is thus essential not only for individual health outcomes but to reduce viral transmission. Failure to maintain optimal levels of adherence is associated with drug resistance and treatment failure. Person with HIV often find it difficult to maintain the adherence necessary for viral suppression due to many factors, including depressive symptoms; [10] even infrequent non-adherence greatly diminishes efficacy [2,11,12].

### Health literacy and adherence in Hispanics

Low health literacy is associated with poor medication adherence in persons with HIV [13,14], but data on health literacy and adherence in SDH are limited. Two studies found limited HL in Hispanic patients with HIV infection [15,16] but did not report on adherence. An educational intervention to improve HL in SDH persons living with HIV (HIV + SDH) was successful in improving knowledge and understanding HIV terms [17], while Levya et al. [18] found comfort in speaking English was associated with better medication dosing. Motivation due to perceived vulnerability and provider support can be instrumental in helping to control the illness through consistent medication use [19]. In addition to HL, cultural aspects of a person’s health beliefs must also be considered as an important influential factor when addressing adherence, yet few studies have been conducted with this group.

### Theoretical framework

The Information-Motivation-Behavioral (IMB) Skills model is an important framework for understanding behavior in those affected by HIV infection. Several studies have supported its validity in HIV-related behavior change [20-23]. The model specifies that health behaviors depend on an individual having knowledge about a problem, motivation to carry out the behavior, and necessary behavioral skills. The usefulness of the IMB Skills model has been demonstrated with Hispanics [24].

We report on a sample of SDH with HIV who participated in a mixed methods study designed to gather data on sociocultural and psychosocial data associated with medication adherence. We present how our findings guided the adaptation of a theory-based, culturally tailored HL intervention to improve medication adherence in HIV + SDH (to be tested in a future study). This intervention extends our previous research on a similar intervention with a multicultural sample of English speakers [25].

## Methods

### Participants

We recruited 25 Spanish-dominant Hispanics with HIV from a community clinic serving the indigent and non-insured Hispanic population of South Florida who met the following criteria: (1) treatment for HIV infection with an antiretroviral medication and stable on that regimen for at least 30 days; (2) able to provide informed consent; (3) 18 years of age or older; (4) self-identify as Hispanic, and (5) Spanish-language dominant. Dominance was determined by responses of participant on the Language Experience and Proficiency Questionnaire (LEAPQ) and observation by bilingual research staff regarding participant’s ability to converse in Spanish versus English. Patients with any psychiatric or cognitive disorder impairing cognition or the inability to provide informed consent were excluded. Approval for this study was granted by the Nova Southeastern University’s Institutional Review Board.

### Recruitment procedures

Members of the medical teams at the clinics approached treatment-seeking patients and determined their interest in learning about the study. Interested persons were then contacted by our research staff for preliminary eligibility screening and to set up an appointment for the one-time interview.

### Quantitative assessment procedures

All interviews were conducted at the referring local community clinic that serves uninsured and indigent Hispanics in Miami, FL. After making an appointment with our staff, recruits arrived at the clinic where a private space was provided. Recruits were then screened by our research staff to confirm eligibility and, if eligible, invited to participate. After giving informed consent, the participants completed a pen-and-paper self-administered questionnaire in Spanish that required 25–30 minutes to complete.

### Measures

#### Demographic items

Seventeen demographic items (e.g., gender, age) were included in the quantitative assessment.

#### Language experience and proficiency questionnaire (LEAPQ)

LEAPQ was used to assess participants’ language-dominance. The 16-item measure is a valid, reliable, effective, and efficient tool for assessing the language profiles of multilingual, neurologically intact adult populations in research settings [26].

#### Marin acculturation scale

This 12-item acculturation scale [27] has been widely used in research with Hispanics. Acculturation, as measured by this scale, correlates highly with respondents’ ethnic self-identification, generation, length of residence in the U.S., and age at arrival (Cronbach’s alpha = .92).

#### The LifeWindows information-motivation-behavioral skills ART adherence questionnaire (LW-IMB-AAQ)

This Likert-type measure (Cronbach’s alpha = .70) assesses barriers to ART adherence within information, motivation, and behavioral skills areas. It also aids in identifying potential adherence related deficits in real-world settings [28]. Seven LW-IMB-AAQ’s information items were used to define ART adherence-related information. Items about personal and social motivation to adhere to their ART regimen were included regarding beliefs about the adverse physical consequences (e.g., side effects) of medications. The measure also includes 14 items targeting adherence-related behavioral skills (Cronbach’s alpha = .88).

#### ACTG adherence baseline questionnaire

This Likert-type measure, developed by the AIDS Clinical Trials Group (ACTG) Recruitment, Adherence, and Retention Subcommittee [29], includes items pertaining to attitude and perception to medication therapy. Items also included were reasons for missing doses in the past month. Participants were asked to identify the last time they missed taking any of their medications (1 = *within the past week*, 6 = *never*). Other items assessed social support, mood/depression, self-efficacy, and alcohol and drug use. Other items asked participants to report on symptoms they had in the past month and level of discomfort for each. Hecht, Colfax, Swanson, and Chesney [30] supported the scale’s validity (e.g., correlation between self-report of nonadherence on this scale with viral load).

### Qualitative assessment procedures

While interview questions were developed to explore themes of interest aligned with the IMB Skills model, our main focus was to explore the phenomenon of individual or sociocultural motivation that might influence adherence to treatment. We enlisted the aid of key informants (e.g., health care providers, pharmacists) who work closely with our target population via informal interviews to help develop interview questions. Phenomenological and constructivists frameworks guided the creation of the semi-structured interview guide. The semi-structured interview guide was translated into Spanish and back-translated to English to ensure linguistic equivalence.

### Qualitative interviews

Qualitative interviews followed by the quantitative survey were conducted by our research staff (trained bilingual interviewers, such as a PhD social worker, a board certified psychiatric clinical pharmacist) who followed an interview guide to explore the following areas: (a) meaning of HIV-related illness (e.g., stigma), (2) cultural context of and motivations for adherence, (c) role of culture in shaping attitudes toward HIV/AIDS and its treatment, and (d) perceptions and misconceptions of HIV illness and treatment.

The participants were asked to discuss the processes by which they decided to take or not to take their medication, about their social support networks, and which cultural aspects of these acted as facilitators or barriers to sustained treatment adherence. These narratives were followed by questions and probes to elicit details. Respondents often spontaneously offered information that was not part of the initial questions. In our prior work, respondents often spontaneously offered information related to one topic area when responding to a prompt from another [31,32]. Thus, although we created a priori sections, they were not rigidly adhered to during interviews according to standard qualitative procedures [33-35]. Participants were given $50 in appreciation of their time. Interviews were digitally recorded and transcribed for coding and cross referencing.

### Data analysis

We analyzed the quantitative data using IBM® SPSS® Statistics statistical software version 20 [36]. Descriptive data (measures of central tendency and standard deviation) were calculated and reported as percentages and frequencies. We used four successive levels of qualitative analysis. We used descriptive analyses to describe the participants’ life situations and characteristics and used thematic analysis to elaborate the structure of the constructs that emerged. We conducted comparative analyses to clarify differences among the participants to ensure our adapted intervention incorporated differences and similarities. After summarizing the concepts and themes that emerged from the qualitative data we used a concurrent triangulation approach [35,37,38] to converge our quantitative and qualitative findings.

## Results and discussion

### Characteristics of the sample

#### Demographics

The characteristics of the sample are depicted in Table 1. Participants’ (N = 25) ages ranged from 29 to 63 (*M* = 47.7, *SD* = 9.72). The sample was heterogeneous in terms of place of origin (i.e., 10 different countries; only 5 participants reported the United States as their place of birth). Forty-four percent had less than a high school education, forty-two percent reported having full or part time work, and 40% reported an annual net income of less than $5,000. Sixty-eight percent were single, the rest were partnered or married.

**Table 1:** **Characteristics of the Sample** (**N** = **25**)

**Variable**	**n**	**%**
Age (mean = 47.7; range = 29-63 years)		
Gender		
Male	17	68
Female	8	32
Education		
Less than high school	11	44
High school diploma/GED	6	24
Some college or vocational school	4	16
Graduate degree	4	16
Employment		
Full or part time	10	40
Unable to work due to disability	7	28
Unemployed	6	24
Annual net income		
Less than $10 k	17	68
Between $10 k–$30 k	5	28
More than $30 k (but less than $50 k)	1	4
Relationship Status		
Single	17	68
Married/Living together	7	28
Paternered/not living together	1	4
Nationality		
Non-US born	19	79

#### Acculturation

The mean score on the Marin Acculturation Scale (Cronbach’s alpha = .93) was 2.2 (lower scores indicating less acculturation [27]. All participants identified as “completely Hispanic” or “more Hispanic than American” and reported Spanish was their first acquired language. The majority (84%) spoke only Spanish as a child; the rest reported speaking some English in addition to Spanish while growing up.

#### Self-reported adherence

About three-fourths (72%) of the participants reported never skipping a dose; 12% reported missing a dose in the past week. Participants reported an array of reasons for missing doses: feeling sick, feeling good, being away, or feeling depressed or overwhelmed.

#### IMB skills for ART adherence

Cronbach’s alpha for the total IMB skills scale was .82, demonstrating good reliability overall. The subscales are described below.

#### Information

(Cronbach’s alpha = .56). The mean score on the Information subscale was 4.2 (1–5 point Likert scale). Most (84%) of the participants reported they are able to take their medications as directed. Some believed skipping a few doses from time to time would not hurt their health. Half of the participants reported they were extremely sure if they did not take their medication as instructed, the HIV in their body will become resistant to HIV medications.

#### Motivation

(Cronbach’s alpha = .72). The mean score on the Motivation subscale was 3.5 (1–5 point Likert scale). Many participants reported feeling sad, lonely or alone, that everything they did was with effort, had problems focusing and/or sleeping, and felt they simply could not continue (in the past week). Some participants reported feeling nervous or stressed (in the past month). Half of the sample reported a dislike for or frustration about having to take HIV medication for the rest of their lives, being constantly reminded they have HIV due to taking doses, having to plan their lives around taking medication, and possible side effects. Fifty percent were worried other people would know they had HIV if they were seen taking HIV medications. Examples of motivation items are listed in Table 2.

**Table 2:** **Examples of quantitative assessment items from the LifeWindows project team LifeWindows Information**-**Motivation**-**Behavioral skills ART adherence questionnaire** (**LW**-**IMB**-**AAQ**)

**Sample items**	**%** **Agree**
I am worried that other people might realize that I am HIV + if they see me taking my HIV medications.	52
I get frustrated taking my HIV medications because I have to plan my life around them.	40
I don’t like taking my HIV medications because they remind me that I am HIV + .	40
I feel that my healthcare provider takes my needs into account when making recommendations about which HIV medications to take.	84
Most people who are important to me who know I’m HIV positive support me in taking my HIV medications.	88
My healthcare provider doesn’t give me enough support when it comes to taking my medications as prescribed.	24
It frustrates me to think that I will have to take these HIV medications every day for the rest of my life.	44
I am worried that the HIV medications I have been prescribed will hurt my health.	56
It upsets me that the HIV medications I have been prescribed can affect the way I look.	52
It upsets me that the HIV medications I have been prescribed can cause side effects.	64

#### Behavioral skills

(Cronbach’s alpha = .85). The mean score on the Behavioral Skills subscale was 4.0 (1–5 point Likert scale). Some participants reported difficulty taking their HIV medications when they drink or use recreational drugs, and they did not know how HIV medications interact with these substances. While some participants had difficulty remembering to take their HIV medications, most found it easy to make them part of daily life. Forty-two percent reported some difficulties in managing their side effects.

### Summary of qualitative findings

Four main themes emerged from the qualitative data: (1) HIV-stigma; (2) social support; (3) mood/depression; and (4) *familismo* (role of family), which are inextricably related. In addition, an overwhelming majority of the participants stressed a preference for Spanish-speaking providers. Participants reported incidents of stigma and discrimination. Many discussed their fear of rejection, verbal abuse, or physical violence. Many participants reported feeling depressed at some point due to having HIV. The most common result of the depression was missed doses. Support, although sometimes difficult to obtain, was also reported as a crucial element in dealing with depression and medications. Many of the participants reported anxiety and concomitant frustration about loss of control (e.g., having to plan their lives around taking medication). Some participants who disclosed their status to family members lost their support. Many participants revealed their greatest medication adherence motivator was the desire to stay alive for their children and/or spouses. Overall, motivational factors including stigma, social support, mood, and family played an important role in the lives of the participants’ ability to adhere to a medication regimen.

This is one of the few studies that examined motivational factors associated with ART adherence in Hispanics with the intent of using them for intervention development. The sample consisted of low-acculturated SDH with HIV that were being treated at a local health clinic serving primarily low income and indigent Hispanics. As would be expected, participants preferred having a Spanish-speaking health care provider. In the case when providers do not speak Spanish, the availability of a Spanish-language tailored computer HL intervention that addresses adherence may be an invaluable tool for physicians’ offices that lack bilingual staff. Although expensive to develop, deployment can be cost effective [39]. Development of these much needed interventions necessitates understanding the intertwining sociocultural and psychological factors contributing to risks for suboptimal adherence and using the findings to guide intervention adaptation as done in this present study.

The mean scores on HIV information and behavioral skills were relatively high on the 5-point IMB Skills subscales (4.2 and 4.0, respectively). However, regarding HIV information, only half of the participants reported they were “extremely sure” that if they did not take their medications as instructed, the HIV will become medication-resistant. Moreover, some participants reported they did not know how HIV medications interact with alcohol or illegal substances. Many were having problems managing the medication side effects.

#### Motivation

The mean score on the motivation to adhere IMB Skills subscale was lower than the information and behavioral skills, which warranted addressing motivation to adhere in a meaningful and culturally relevant way. Motivation to adhere to HIV medication has been thought to be influenced by sociocultural (e.g., stigma, social support) and psychological factors (e.g., depression).

#### Stigma

Of relevance to intervention adaptation is that stigma was a factor in medication adherence among our sample. Participants reported multiple incidents of enacted stigma and discrimination. Many discussed their fear of rejection, verbal abuse, or physical violence. Secrecy is such an issue for these patients that if they see someone they know in the clinic who has HIV, they will go to another clinic. These patients live a secret life. Their family and children know they have something, but are told it is some medical condition like a more acceptable condition (e.g., hypertension, cardiac abnormality, cancer). The families may be supportive but are unaware it is HIV. Overall, there was the overwhelming concern other people would know they had HIV if they were seen taking their medications. These findings are supported by studies on Hispanics indicating they experience high levels of HIV stigma [40]. Societal intolerance of HIV and stigma-related experiences can affect medication adherence in Hispanics with HIV [41].

#### Social support

What emerged related to social support is also noteworthy. For Hispanics from countries that have had governmental discriminatory practices (i.e., confining HIV patients to hospitals), their trust for medical providers may be compromised. Because SDH may experience high levels of HIV-related stigma, HIV-positive Hispanics may experience an even greater loss of social support, higher levels of persecution, and isolation [42]. Experiencing stigma from family and/or consequent lack of disclosure from HIV-positive people in family settings may result in greater reliance on non-kin for social support [43].

#### Mood/depression

Many participants reported feeling depressed at some point due to having HIV. The most common result of the depression was missed doses. Support, although sometimes difficult to obtain, was also reported as a crucial element in dealing with depression and medications. Our findings are supported by studies that show depression is among the most common neuropsychiatric disturbances seen in persons with HIV infection. It has an impact on the course and outcome of HIV infection through its effects on patients’ adherence to medication regimens via cognitive slowing, lack of motivation, and feelings of helplessness [10]. A pilot study of Hispanic men living with HIV found 65% participants to be depressed [44]. Another study found 66% of Hispanic women living with HIV scored above the conventional threshold of possible clinical depression [45]. A trial with Hispanic women with HIV found depressive symptoms and suicidal ideation were common [46]. Several studies have found that limited English proficiency is a significant barrier to health care access and quality care. Moreover, Spanish-speaking Hispanics are significantly less likely than majority whites to make a physician or mental health visit [47].

#### Familismo

To more fully understand adherence in HIV + SDH it is important to consider the environmental context of family and culture. Some participants who disclosed have lost the support of family members due to their HIV status. Nonetheless, family played an important role in the lives of the participants. Many participants revealed their greatest medication adherence motivator was the desire to stay alive for their children and/or spouses. While Hispanic culture is by nature a communal one, with different generations and extended family members sharing one household, isolation and secrecy are survival techniques used by some Hispanics with HIV. These issued combined with the aforementioned factors can make medication adherence challenging to Spanish-dominant Hispanics.

### Limitations

There are several limitations that should be considered. First, the data are cross-sectional and do not provide information on cause and effect or changes over time. Second, although the sample is heterogeneous in terms of country of origin, our small sample is not necessarily representative of the broader population of SDH living with HIV. Rather, our generalizability is for the purpose of intervention adaption only (to be tested in a larger study) with limited data gathered from inner-city low income HIV + SDH. Last, many of the participants had been taking their medications with regularity; complications regarding missed doses or non-adherence was from past, not current, experiences of participants; recall may not be accurate.

### Development of the intervention

#### Technology

Content for the one-hour intervention was initially developed by a multidisciplinary team that included representatives from medicine, social work, pharmacy, psychology, and nursing [48,49]. The content was organized into sections focused on basic HIV-related information (e.g., viral life cycle and mechanism of action of medications), factors and possible barriers related to motivation (e.g., misconceptions about medications and strategies for coping with stigma, depression, and substance abuse), and behavioral skills (e.g., strategies to remember to take medications). Material was presented in short passages of text supported by pictures, illustrations, and an animation supplemented with audio narration. These were followed by assessing participant understanding through multiple choice questions. When a participant failed to answer an assessment question correctly, the material was immediately retaught after displaying a personalized message including the participant’s name, such as “That’s not quite it, María, Let’s go over that again.” Such computer interactions required participants simply tap on the computer screen, requiring minimal computer experience. Overall, these touch screen computers are highly accepted by users in healthcare and research settings with persons living with HIV [50-58]. The published application can be deployed on the Internet and accessed in a browser or installed as a free-standing application on a computer.

### Linguistic and cultural adaptation

Adaptation of the HL intervention to increase medication adherence in HIV + SDH was guided by the findings presented in this article as well as those previously published or currently under review. At the core of the adaptation is the recognition of the intertwined nature of motivation and the importance of addressing linguistic and sociocultural barriers and facilitators that influence motivation to adhere to a medication regimen. An important component of the intervention is to help participants understand how culture, family, social support, and stigma influence their ability to adhere to treatment.

### Linguistic component

The development of the adapted intervention entailed linguistic translation of the actual computer application using the procedures described by Marin and Marin [59]. The Spanish version was back translated into English. We adapted the current English-language intervention to protect the integrity of the intervention while ensuring it is understood in Spanish. The translation and adaptation effort utilized a process of successive review and revision of the working drafts of the intervention, as suggested by Stansfield [60] and previously conducted in the field by members of our research team in previous studies [32,61-65]. Final decisions on content were made by consensus of the investigative team.

### Cultural motivational component

#### Stigma

In this section participants are reminded that they must take their HIV medications 95% of the time, which we mention could be very difficult to do. We validate a very real concern that some people (e.g., family, co-workers) may find out they have HIV and might even be disgusted or angry with them. They might be thought to be a drug addict or prostitute or other types of people who are negatively associated with HIV, and not accepted in the Hispanic community. We offer suggestions for constructive ways to manage thoughts and feelings of rejection or the fear of rejection. For example, one screen states: Some people may worry that other people know they have HIV. Some people are very critical about people with HIV. Some people who have HIV worry that other people will find out. [Name], would you like to know more about how to handle this problem? (yes or no). If they answer yes, the intervention continues with cognitive exercises to help the participant work through their thoughts and feelings, and then offers suggestions.

Many of the participants had body image issues and concerns of the physical changes associated with HIV medications. We thus deemed it necessary to add a portion on side effects that dealt with physical changes that sometimes occur when taking antiretroviral medications. The English version discusses what to do if you experience symptoms of side effects (i.e., consult a health care provider), but based on our findings we determined it would be important to address the physical changes that might draw attention to their HIV-status which is linked to the stigma. Here is where we introduce the concept of seeking social support via support groups.

#### Social support

The intervention addresses with importance of finding social support to combat the isolation, loneliness, fears of or actual rejection from loved ones. We discuss the tendency to feel sad or withdraw from usual social scenes and society at large. We end this section with suggestions of seeking support in the form of friends, family, or an HIV support group. Involvement in social support groups has been associated with higher self-reported adherence, higher social support, and lower depressive symptomatology [10].

#### Familismo

Emotional support from family and friends consists of providing encouragement to take medications, attend appointments, and follow up on medical care. The Hispanic family, traditionally the most important social unit, tends to go beyond the nuclear family. The term *familia* usually includes parents and children but also extended family members. While there is generally a moral responsibility to help other members who may be experiencing financial problems or poor health conditions, some members living with HIV may not be cared for in the same way due to the stigma attached to the disease. Considering the central role of the family in Hispanic/Latino cultures, this can be problematic for Hispanics with HIV [66]. It is here where we expand on the idea of turning to friends and other trusted individuals (e.g., priest) as well as HIV support groups. In this case, non-kin or self-created families can emerge.

#### Mood/depression

In this section we approach the idea that even when you feel bad you should remind yourself that you still need to take HIV medication as prescribed. Also introduced is the notion that if you are depressed, you should talk to someone, such as friend, pharmacist, priest, or therapist, and there are medications for depression that might help. We add the possibility they may feel like a bad person for having HIV, but they do not have to feel ashamed. We also address the notion that some people have a problem with alcohol or drugs, which can not only impair adherence but can compromise their immune system, too. Again, we attempt to motivate the viewer to seek help from a health professional when warranted.

### Future study

In the next phase of this research we plan to test the intervention whereby participants recruited from local health clinics will complete three visits after randomization to either the HL and adherence intervention or an attention control educational application. In addition, participants will be oriented to the use of the Medication Event Monitoring System (MEMS) that will be used to monitor their adherence. Adherence and other potential mediators of adherence behavior will be assessed on a final visit.

## Conclusion

A promising strategy for providing HL interventions to improve medication adherence for Spanish-dominant patients in busy or limited-resource settings is to provide automated computer-based applications. Information technology-based interventions promoting HL and medication adherence can be available on computers, tablets, or via the Internet. Once created, costs for their continued maintenance and deployment can be low, making them an inexpensive strategy for addressing HL and adherence issues in persons with HIV [39,67]. Further, research indicates motivation is instrumental in helping to control the illness through optimal adherence [68]. Our approach integrated the established usefulness of the IMB Skills model and tailored information interventions within the general rubric of HL to produce a culturally-relevant computer-based automated intervention that can readily be deployed at lost cost and with minimal demands on clinician time. It is a synthesis of instructional, computer, cultural, and biomedical research applied to a critical problem in caring for HIV + SDH patients.
